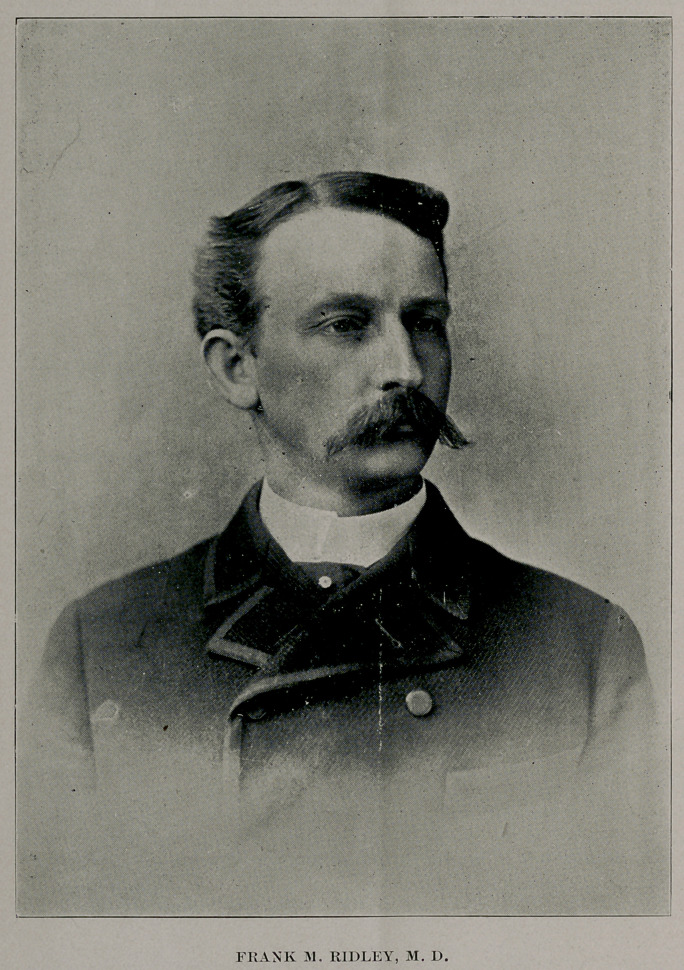# A Duty of the State Medical Association

**Published:** 1895-05

**Authors:** F. M. Ridley

**Affiliations:** LaGrange, Ga.


President Medical Association of Georgia.

THE

### Southern Medical Record.

A MONTHLY JOURNAL OF MEDICINE AND SURGERY.

VOL. XXV. ATLANTA, GA., MAY, 1895. No. 5.

### Original Articles.

### A DUTY OF THE STATE MEDICAL ASSOCIATION.*

BY F. M. RIDLEY, M. D., LAGRANGE, GA.

Were I disposed to indulge in mere verbal criticism I might say that the term lunatic, as applied to the victims of mental disease, whether organic or functional, is palpably misleading. So much so, indeed, that it deserves to be banished from our medical nomenclature, nor less does it deserve to be expunged from our school dictionaries. Its current use grew out of the venerable superstition that "The inconstant moon, which nightly changes its circling orb," is an important factor in the production of nervous disorders, and that its various phases have great influence on their successful treatment. This theory of the ancient astrologists is no longer accepted outside of extremely rural districts where a belief in ghosts and witches still has a foothold in the popular creed.

The general term insanity is far more significant, and has in recent years utterly exploded the idea of lunatic influence in the production of all classes of neurotic ailments.

The word embodies a conception of the most direful malady that has ever afflicted the human race since Adam and Eve were driven from Eden, and the tree of life transplanted to that Apocalyptic river where "it grows twelve manner of fruits, and whose leaves are for the healing of the nations."

*Read before the State Medical Association, Savannah, Ga., April 15, 1895.

If our statistical tables may be relied upon, the increase of this fearful disease may well alarm the philanthropist and the physician.

How much of this is due to our sanitary methods and our habits of imprudent living is in no small degree conjectural. Heredity, we know, plays an important part in this tragic drama, and social customs, notably among these, the morphine and whiskey habits, and the careless use of narcotics in the hands of physicians, have contributed to swell the ranks of the vast procession that journeys on to degradation and death. Fortunately there has been developed in these later years, along with skillful diagnosis, a wonderful method of brain surgery, which in many organic lesions of the great nerve center, has wrought wonderful results.

But what is not less a matter of congratulation is the vast strides which have been made since the early days of Bedlam and Bicetre, in the treatment of functional neuroses by electric agencies, by scientific massage, and by intelligent medication. These systems will inevitably lessen the ratio of mortality, and diminish the alarming increase of insanity to which I have already referred. Vast improvements in this direction accumulate where proper attention is given to the moral treatment and proper dieting of the inmates of our numerous State asylums.

Another step brings us to the consideration of the proposition suggested in the title of this paper. It is a question which profoundly concerns the members of this association.

You, gentlemen, will readily acquit me of any purpose to question the personal integrity or professional ability of that cultured gentleman who, during a long series of years, has held the superintendency of the State asylum, and who, in connection with his assistants, has conducted its therapy and its discipline, but it would be disingenuous in me to deny that there has been dissatisfaction at conditions, and disappointment as to its practical results.

Under any management mistakes are inevitable, but it is none the less our duty, as I conceive it, to, as far as in our power lies, correct them and remove the cause of them, if we are wise enough for the task.

Most, at least, of us are satisfied that inadequate legislative appropriations to a great extent lie at the bottom of the evils to which we allude.

According to one of the latest reports of Dr. Powell, there are now about 1,800 inmates in the asylum. So densely packed are these unfortunates that fresh applicants are frequently of necessity refused admission to the place on the ground (which is anything but a shallow pretext) that it would be dangerous to allow the number of patients to increase. As a consequence, in a deplorable number of instances, filthy county jails, swarming with vermin and poorly furnished with beds and covering, are converted into temporary sanitariums for the treatment of the insane, and this, too, at the outset of the attack, when skillful nursing and the best medication are most demanded.

Into these wretched prisons, provided for the safekeeping of thieves and culprits, are thrown delicate women and gray-haired sufferers, not for crime, but on account of sheer misfortune. Surely every Georgian not devoid of becoming state pride feels the blood mantle his cheek for shame as he gazes on this not overdrawn picture.

We do not charge these things on Dr. Powell or his Board of Trustees.

We have witnessed with what assiduity he has plied the Georgia legislature, year after year, with arguments and appeals for funds to be appropriated to the amplifying and furnishing the asylum buildings. By untiring persistence an appropriation quite too small was secured. The work has been singularly delayed, however, by whose default we are not advised. But it is now believed that the new structure will be in readiness by the close of the year. Let us hope that something will be done by which no emergency such as the present will ever again overtake this institution. As a means of preventing this it has been suggested that a new asylum should be promptly erected at Atlanta, Augusta, Savannah, Griffin, or some other eligible point, for the exclusive occupancy of the colored race. This plan is both practicable and desirable. Before the civil war insanity was a rare disease among the negroes of the South. The emancipation policy which Mr. Lincoln precipitated on the country, avowedly for humanitarian purposes, but really to secure himself a second presidential term, has imposed a heavy burden on the taxpayers of Georgia, who have to educate their own children and provide shelter, treatment and subsistence for their pauper insane.

This is an obvious necessity and a part of our heritage, resulting from the issue of the late war. These "Wards of the Nation," God save the word, are put under restraint for their own security as well as the protection of society. In this, as well as in our schools and churches, it is needful to draw the color line, if we would avoid bickerings and antagonisms.

Another change is demanded in regard to the visitation of the asylum. The present plan is in many respects objectionable. Hitherto a committee has been dispatched, at stated intervals and at the public expense, to investigate the affairs of that institution. It is composed of laymen, generally, who know as little of insanity as they do of Sanskrit. The officials of the asylum, of course, know of their coming, and consequently, naturally and properly, everything is put on the best footing possible for their arrival. In due time their report is submitted to the legislature and goes through that body without a call for the yeas and nays.

Suppose another scheme of investigation should be inaugurated, *i.e.*, let the governor appoint a committee composed of seven of our ablest physicians to visit the asylum at least once a year and not at stated times. Let them be clothed with full powers to make a searching, sifting examination of every department, from the kitchen to the superintendent's office. Submit to his excellency a carefully prepared report to be transmitted by him to the legislative body, suggesting such improvements in sanitation and general conditions as may seem to them to be urgent and feasible. Let them urge greater attention to the moral treatment of the inmates, as may be suggested by expert study and investigation of the best systems of Europe and America.

This, it may be said will, require some outlay of money. I would suggest that money thus spent is well-spent, and should be met by the funds of the State treasury. I forbear further details. If this intelligent body sees proper to take hold of this important matter, and I believe it will, the commission appointed could accomplish all I have suggested, and more.

One thing that has been alleged against the present management is the improper classification of patients. Superintendent Powell in his last annual report to the Honorable Board of Trustees, admits the correctness of this view, and attributes it to the overcrowded condition of the buildings. This should be properly investigated, as it is, of necessity, a sore evil. It is obvious that nothing is more likely than this unfortunate condition to prevent and retard the convalescence of the inmates. Frequent contact with persons with whom they have no moral affinity and no community of tastes is sure to arouse the resentment of any class of the insane.

Superintendent Powell suggests that it might occasionally lead to acts of violence that would mar the harmony and discipline of the institution.

Too much care cannot be exercised in the choice and assignment of capable attendants to particular male and female wards. If there is a misfit in these details, the attendant is quite sure to be a hindrance rather than a help to the recovery of the patients. Indeed, within the narrow scope of his duties, the attendant is but little less important than the physician. Every care is necessary in the selection of these subordinate officials. Cheapness should not be regarded in comparison with fitness. It is likewise of prime importance that ample provision should be made for placing its surgical department on a basis with all the improved appliances, with all the advanced skill of the present century. In latter days wonderful strides have been made in brain surgery. The State asylum should be in thorough equipment for its successful practice.

There is another department of the work of the asylum that claims our attention and which calls for the careful consideration of this association. I refer to the inadequacy of the medical staff to perform the vast amount of professional labor assigned it. As previously stated, there are about 1,800 inmates, suffering a score or more of nervous ailments. For the medical treatment of this multitude there are not exceeding five on the medical staff, a ratio of 600 patients to one physician, proportionally a greater charge on the physicians of the Georgia

Insane Asylum than of any other similar institution, not on y of the United States, but of the world.

A very simple arithmetical calculation demonstrates their insufficiency to do with efficiency the work required of them.

It occurs to me, Mr. President and gentlemen of the association, that it would not be out of place to memorialize the governor to co-operate with a committee from this association looking to the remediation of these conditions.

I am quite sure that all and singly, the suggestions of this paper will meet the approval of Dr. Powell and his assistants, who have doubtless already had them in their thoughts. Let us hope that the present session of this intelligent body, convened from all parts of the State, will give fresh impetus to the asylum.

We owe it to ourselves, we owe it to humanity, nor less to the State, to bring ourselves in touch with an institution established for the benefit of a class who are literally powerless for their own protection.

Who are so well-fitted as the members of the medical profession to aid and direct in this work of humanity, as you, gentlemen; who have so often braved the storm and the frost, administering alike to the rich and the poor, whether in their palatial homes or dingy hovels?

Every student of Shakespeare has been struck by the incidents of that midnight tempest which beat so ruthlessly upon the unsheltered head of King Lear, crazed by the cruelty of his unnatural daughters, Reagan and Goneril, wandering aimlessly over the "blasted heath" while the lightning's red glare fitfully illumined the tragic scene and the deep-toned thunder "rumbled its belly full;" and yet that other scene where "my Lord Hamlet," having seen the ghost of "buried Denmark," argues with himself that question "to be, or not to be." Such spectacles of frenzy are of nightly occurence in the Georgia asylum. And they appeal loudly to every sympathy for help and for healing.

But let the curtain fall upon this gruesome picture. Let us, gentlemen of the association, lend our undivided and united aid to the betterment of these conditions, to the improvement of our insane asylum, and thus, in a larger measure than ever before, serve our fellow-man by thus contributing to the alleviation of suffering humanity.

## Figures and Tables

**Figure f1:**